# COVID-19 and Nepal: Identification of Critical Public Health Measures

**DOI:** 10.31729/jnma.4950

**Published:** 2020-05

**Authors:** Harish Chandra Neupane, Niki Shrestha, Shital Adhikari, Basanta Gauli

**Affiliations:** 1 Department of Surgery, Chitwan Medical College, Bharatpur, Chitwan, Nepal; 2 Department of Community Medicine, Chitwan Medical College, Bharatpur, Chitwan, Nepal; 3 Critical Care Division, Chitwan Medical College, Bharatpur, Chitwan, Nepal

**Keywords:** *COVID-19*, *Nepal*, *World Health Organization*

## Abstract

The COVID-19 pandemic is unfolding at an unprecedented pace. The unprecedented threat provides an opportunity to emerge with robust health systems. Nepal has implemented several containment measures such as Rapid Response Team formulation; testing; isolation; quarantine; contact tracing; surveillance, establishment of COVID-19 Crisis Management Centre and designation of dedicated hospitals to gear up for the pandemic. The national public health emergency management mechanisms need further strengthening with the proactive engagement of relevant ministries; we need a strong, real-time national surveillance system and capacity building of a critical mass of health care workers; there is a need to further assess infection prevention and control capacity; expand the network of virus diagnostic laboratories in the private sector with adequate surge capacity; implement participatory community engagement interventions and plan for a phased lockdown exit strategy enabling sustainable suppression of transmission at low-level and enabling in resuming some parts of economic and social life.

## INTRODUCTION

The World Health Organization (WHO) was alerted when several cases of pneumonia with unknown etiology were found in Wuhan City, Hubei Province of China on 31^st^ December 2019.^1^ On 2020 January 7, Chinese authorities confirmed the cause of pneumonia was due to the novel coronavirus.^2^ On January 30, 2020, Public Health Emergency of International Concern (PHEIC) was declared by WHO.^3^ To avoid confusion and facilitate communication, on 2020 February 11, WHO named the virus as SARS-CoV-2 and named the clinical illness as COVID-19.^4^

## EVOLVING GLOBAL SITUATION

**Figure 1:**
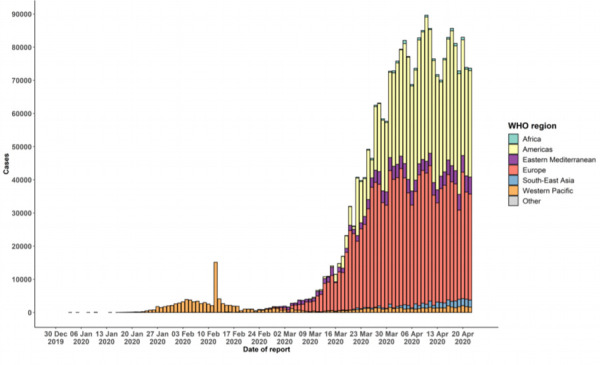
Epidemic curve of confirmed COVID-19, by date of the report and WHO region through April 23, 2020.^6^

With an increasing number of cases in China and spread to other countries, WHO declared COVID-19 transmission as pandemic on 2020 March 11.^5^As of April 23, 2020, a total of 2,544, 792 people have been confirmed with COVID-19 worldwide and a total of 1,75,694 deaths have occurred from COVID-19.^6^ In the South-East Asia Region, there have been a total of 36,039 confirmed cases of COVID-19 and 1,498 deaths.^6^ In Nepal, 47 cases have been diagnosed with more than two-thirds from Udaypur district.^7^ The WHO risk assessment at the global level at present is “very high” (Figure 1).^6^

## NEPAL’S RESPONSE

A Nepalese citizen returning from Wuhan City on 2020 January 9 was tested positive for COVID-19 on 2020 January 23 by RT-PCR when his throat swab was sent to Hongkong for laboratory testing.^8^

Soon after, all the hub hospitals and key satellite hospitals in Nepal were asked to identify a dedicated space with isolation facilities for COVID-19 cases.^8^ Technical documents were prepared by the Ministry of Health and Population (MoHP) for investigation and containment of COVID-19.^8^ Tribhuvan International Airport installed a Thermal Scanner and the National Public Health Laboratory (NPHL) was capacitated for polymerase chain reaction (PCR) testing from a respiratory specimen from 27 January 2020.^8^ A high-level technical team was formed to monitor the situation across the hub and satellite hospitals network.^9^ Also, a regular communication mechanism was established between Health Emergency Operation Centre (HEOC), Provincial Health Emergency Operation Centre (PHEOC) and Ministry of Social Development (MoSD).^9^

To prepare for the possible outbreak of COVID-19, information, education and communication (IEC) materials were developed and disseminated at strategic locations across the country as well as through Television and Radio.^10^ Safety of health care workers (HCWs) was planned to be ensured with the provision of personal protective equipment (PPE).^10^

Health desks were established at the provincial levels.^11^ Hospitals were directed to prepare their Hospital Disaster Preparedness and Response Plan.^11^ The daily reports of suspicious cases were started to be sent to the ministry.^11^ Hospitals were directed to comply with the algorithm and protocol prepared by the MoHP.^11^ Provincial Health Emergency Operations Centers (PHEOC) started working in close coordination with the ministry.^11^ For the convenience to the public; hotline service was started to provide information on COVID-19.^11^

Looking at the imperative need to train health workers, the National Health Training Center (NHTC) initiated orientation training to 86 health workers who would be involved in the quarantine and screening for COVID-19.^12^ As early as Feb 13, 2020, orientation on contact tracing and use of Personal Protective Equipment (PPE) was started in the country.^13^ Resource mapping of identified Hub & Satellite Hospitals Network were carried out and simulation exercises were conducted for responding to COVID-19.^14^

The Department of Health Services (DoHS) mobilized its team members to each of the provinces for monitoring the situation and further planning with an emphasis on strengthening of health desks, hospital management and surveillance system.^15^ As an additional effort to combat COVID-19, orientation programs were carried out for community schools, schools under Private & Boarding Schools Organization Nepal (PABSON), and National-PABSON.^16^

The algorithm to suspect COVID-19 infection based on the WHO Surveillance "Case Definition" was prepared by the country and hospitals were identified in various provinces for the establishment of isolation wards, isolation ICUs equipped with ventilator facilities and a definitive budget plan was formulated for the same.^17^ Also, the “Protocol for Health care facilities for COVID-19 case Management” was prepared and surveillance officers were appointed for active surveillance.^18^ Different committees for coordination at different levels- international, national, provincial and local for drug management, supply management, laboratory management, information management and staff management have been appointed for the more effective management of COVID-19. Similarly, high-level directives committee, provincial coordination committee, various subject committees, and special task force committees including rapid response team (RRT) have been created in various Provinces for control and response.^18^

As part of the containment strategy suggested by WHO, the lockdown was started in Nepal from Chaitra 11, 2076 (March 24, 2020).^19^ A COVID-19 Crisis Management Centre (CCMC) on 31^st^ March 2020 was established in the country under the High-Level Coordination Committee on Prevention and Control of Novel Coronavirus under the leadership of Honorable Deputy Prime Minister and Defense Minister.^20^ CCMC was established to deliver rapid responses to COVID-19 in an organized, effective and coordinated manner. The CCMC will also monitor the supply of essential medical equipment, health materials and maintenance of peace and security in addition to conducting necessary activities for prevention, control and treatment of COVID-19.^20^

On 30th Chaitra, 2076 (12th April 2020), an “Interim guideline on COVID and other health care services in the context of COVID-19 pandemic - 2076” was released.^21^ Efforts have been made to increase the scope of testing, effectively executing contact tracing, and strictly follow the lockdown. Discussions were held on developing short-term (emergency), mid-term and long-term strategies. The public health experts have reaffirmed their commitment in the fight against COVID-19 pandemic.^22^

About the “Interim guideline on delivery of COVID and other health care services in the context of COVID-19 pandemic-2076”, 16 private hospitals in Kathmandu valley have been added to provide services through COVID-19 clinic in addition to 25 hub hospitals, 64 provincial hospitals and 22 medical colleges.^23^ Under the leadership of the coordinator of the team mobilized by the Ministry of Health and Population, a province-level committee has been formed. The purpose of this committee is to facilitate coordination between the federal, provincial and local level. The committee will also be involved in the management of human resources and testing as well as the implementation of COVID-19 related activities in the provinces.^23^

Epidemiology and Disease Control Division (EDCD), has trained several public health professionals on case investigation and contact tracing.^24^ Standard Operating Procedure (SoP) has been prepared for COVID-19 hospitals and clinic.^25^ As of 22^nd^ April 2020, 15 PCR laboratories were established across the country.^26^

## WAYS FORWARD

The COVID-19 pandemic is unfolding at an unprecedented pace. Though we are getting new information about COVID-19 almost daily, many things are to be known about the natural history, incubation period, modes of transmission, the period of communicability and case fatality rates.

To respond to the ongoing pandemic, identification and continuous implementation of critical public health measures and significant investment in those measures are of the utmost importance.

First, as recommended by the World Health Organization, the national public health emergency management mechanisms should be activated with the proactive engagement of all the relevant ministries such as health, education, agriculture, public works, travel and tourism, environment, social protection, etc. to provide coordinated management of COVID-19 preparedness and response.^27^

Second, the enhanced focus should be placed on early action and implementation of comprehensive public health measures- such as rapid case identification, rapid testing and isolation of cases, comprehensive contact tracing and quarantine of contacts because these measures help suppress the spread of COVID-19 below the threshold at which health systems become unable to prevent excess mortality.^28^

Third, emphasis must be laid on a strong, real-time national surveillance to detect COVID-19 cases and measures should be applied to prevent an outbreak and if needed, immediate containment measures should be in place.

Fourth, community transmission in Nepal looks imminent and we need to show our preparedness by focusing on capacity building of a critical mass of health care workers to ensure skilled and adequately trained human health resources at Federal, Provincial and Local levels.

Fifth, we need to further assess infection prevention and control (IPC) capacity at all levels of our healthcare system, including public and private and at federal, provincial and local levels. The minimum requirements include functional triage system and isolation rooms, trained staff (for early detection and standard principles for IPC); and sufficient IPC materials, including WASH services/hand hygiene stations and personal protective equipment (PPE).^27^

Sixth, good laboratory practices that produce accurate results are integral to assure that laboratory testing benefits the public health response. Nepal can expand its network of virus diagnostic laboratories in the private sector with adequate surge capacity and we must move from the rapid diagnostic test (RDT) to exclusively the RT-PCR tests only, as recommended by WHO.^29^

Seventh, communities must be empowered and the services ideally should be planned and implemented based on the community’s feedback. Only with the support of motivated people from the community, can we enhance our focus on critical functions such as community education, supporting health workers, protecting vulnerable groups, case finding, contact tracing, and cooperation with physical distancing measures, hand hygiene and respiratory etiquette. Research needs to be undertaken to understand the knowledge, attitude, perception, behaviours of the community and thereafter, identify the proper channels and community-based networks to promote scientific and public health messages for an effective response to COVID-19 pandemic. Participatory community engagement interventions should include accurate information on risks, what is still unknown, what is being done to find answers, what actions people can take to protect themselves and what actions are being taken by health authorities.^28^

Eighth, our focus must also be on displaced populations, migrants and people residing in high-density and informal settlements, because they are at particularly high risk from the interruption of already limited health and social services.^28^

Last but not the least, as recommended by WHO, there is an urgent need to plan for a phased lockdown exit strategy that will enable the sustainable suppression of transmission at a low-level as well as enable in resuming some parts of economic and social life. The phased transition from lockdown should focus on carefully balancing socio-economic benefit and epidemiological risk. The lack of adequate and cautious planning and the simultaneous absence of scaled-up public health and clinical care capacities while prematurely lifting the physical distancing measures may lead to an uncontrolled resurgence in the transmission of COVID-19 as well as an amplified second wave of cases.^28^

With the world facing an unprecedented threat, there is an opportunity to emerge with stronger health systems. There is a need for increasing testing capacity, contact tracing and isolation and quarantine to contain the disease. At the same time, we need to focus on creating robust and resilient health systems and mobilizing all sectors and communities to ensure that every sector of government and society takes ownership and participates in the response in tackling the COVID-19 pandemic.

## Conflicts of Interest:

**None.**
